# Emergency Department-Based Medication for Opioid Use Disorder Program: Addressing Gaps in Linkages to Care

**DOI:** 10.7759/cureus.62927

**Published:** 2024-06-22

**Authors:** David C Seaberg, Jamie McKinnon, Lyn Haselton, Quentin Reuter, Jason Kolb, Suman Vellanki, Nicholas Jouriles

**Affiliations:** 1 Emergency Department, Summa Health, Akron, USA; 2 Psychiatry and Behavioral Sciences, Summa Health, Akron, USA; 3 Research, Summa Health, Akron, USA

**Keywords:** addiction therapy, behavioral health, substance use disorder, adult emergency department, medication for opioid use disorder

## Abstract

Background

Emergency department (ED)-based medication for opioid use disorder (MOUD) has been shown to be effective in providing ease of access and successful treatment rates for patients with opioid use disorder (OUD). This study examined the social determinants of health (SDOH) of patients entering an ED-based MOUD program through individual and focus group surveys. SDOH may impact treatment retention for current and future patients.

Methods

A survey of all patients entering our MOUD program at two hospital-based EDs and two free-standing EDs was conducted from January to March 2022. Addiction care coordinators (ACCs) used standardized screening tools to enroll patients into the MOUD program, and trained research coordinators used a standardized form, using previously validated survey questions, to examine the role of SDOH. Focused group surveys were also collected. The survey measured patients’ perspectives of the program and solicited feedback on SDOH and program barriers.

Results

Of the 60 OUD patients inducted into the ED-based MOUD program during our survey period, 19 (32%) participated in an individual or focus group interview. Of these, 16 patients (27%) completed all survey questions. The mean age was 42 years old, 94% identified as Caucasian, and 65% were males. Over 94% of subjects found the ACCs helpful in providing follow-up care. Nearly 40% experienced transportation and financial issues. The vast majority found the MOUD program beneficial in coping with withdrawal symptoms, dealing with their addiction, and supporting recovery.

Conclusion

OUD patients found the ACCs and the MOUD program helpful for their transition to the treatment stage. The MOUD program can improve some patients’ reluctance to engage with a healthcare system by addressing barriers related to transportation to appointments and financial issues.

## Introduction

The United States has seen a significant increase in prescription drug misuse, leading to the opioid crisis. In 2020, an estimated 2.7 million people in the United States had an opioid use disorder (OUD) [1]. The annual OUD-related costs to the US were 86.8 billion in 2018 [2]. Despite the attention OUD has received, opioid deaths have increased significantly four-fold over the last 12 years [3]. To improve access to treatment for those with OUD, emergency department (ED)-based medication for opioid use disorder (MOUD) programs have been shown to be successful in treating this disorder [4,5].

Social determinants of health (SDOH) are critical elements related to OUD health outcomes. SDOH are non-clinical factors, including the conditions in which people are born, grow, live, work, and age [6,7]. Addressing SDOH and improving health care are often interrelated and conflicting priorities [8,9]. Difficulty procuring employment, transportation, or housing, for example, may pose immediate threats to well-being, making seeking health care a lower priority [9].

Given the substantial impact SDOH have on MOUD patient outcomes, this study sought to clarify which SDOH are most impactful in our OUD population utilizing individual and focus group surveys. Additionally, an examination of our addiction care coordinators' (ACCs) effectiveness in improving SDOH and well-being was measured. The aim of this study was to examine the SDOH of patients entering an ED-based MOUD program through individual and focus group surveys.

This article was previously presented as a meeting abstract at the 2023 American College of Emergency Physicians Scientific Assembly on October 9, 2023.

## Materials and methods

Program description

Summa Health's (SH) First Step program is an innovative ED-based MOUD treatment program for patients with substance use disorder (SUD)/OUD. It uses the Screening, Brief Intervention, and Referral to Treatment (SBIRT) evidence-based model [10] to provide treatment and features ACCs, who are registered nurses focused solely on SUD patients. ACCs offer routine nursing services, care coordination, and individualized treatment plans based on the American Society of Addiction Medicine (ASAM) guidelines. They address comorbid conditions, facilitate transitions to internal and external services, and connect patients to recovery support services (RSS), including peer recovery specialists (PRS) from Catholic charities. Harm reduction efforts include referrals to needle exchange programs and distribution of naloxone kits. ACCs were available daily from 11:00 am to 11:30 pm at Barberton and Akron city EDs and provided outreach and follow-up to support patient recovery. The program has expanded to two free-standing EDs via telemedicine with support from an Ohio Department of Mental Health and Addiction Services (ODH) grant.

Study design/intervention

This was a prospective convenience sample of ED patients enrolled in an ED-based MOUD program between January and March 2022 at two hospital-based EDs and two freestanding EDs. The three-month time period was defined by the ODH grant. Patients aged 18 years and older were eligible for enrollment into our MOUD program if they had OUD and a clinical opioid withdrawal score (COWS) greater than or equal to eight. Since this was part of a quality improvement project, the Summa Health System Institutional Review Board (IRB) found it to be exempt from review; therefore, there is no IRB number associated with it.

We used trained researchers to ask our patients for focus group feedback to address gaps in linkage to care for Summa’s ED-based MOUD program. The goal was to ascertain from our patients their perspective on the program and solicit feedback on social determinants of health and barriers encountered in the program. We used the feedback to make changes to improve the program. Each patient enrolled in the MOUD program was called by phone at their last known contact number. They were asked templated, previously validated questions about their experience, and the results were tabulated. Each patient was also invited to participate in a group interview.

The program held two live focus groups in early 2022, as well as an individual program feedback survey. Participants were contacted regarding their interest in providing feedback on their experience with the program via a virtual (WebEx) focus group format or completing a feedback survey over the phone with a trained research coordinator or emergency physician.

During the live WebEx focus groups, discussion among participants was facilitated, and the questions included were to gain insight into the participants' experience with the program and how it could be improved. During the WebEx and telephone surveys, information was collected on satisfaction with a virtual consultation and telemedicine options, ability to cope with withdrawal symptoms, issues contacting study coordinators, if they felt their treatment plan was effective, and whether they were well supported, and their sense of empowerment over their lives.

Measures

Previously validated metrics from the Health Opportunity and Equity (HOPE) Initiative [11] and State Health Improvement Plan (SHIP) [12] were used, as required by the ODH grant, to develop questions for the standardized survey for the individual interviews and focus group survey. Focus group surveys concentrated on those SDOH that affected care and recovery in our MOUD program. The survey concentrated on mental health recovery questions, social and economic factors, as well as access to psychiatric care. Additionally, we inquired whether virtual services were helpful. A median income by zip code analysis was performed using the US Census Data to compare our survey participants to the surrounding area [13].

Using a standardized questionnaire process, patients were asked about their perspective on the program, SDOH barriers encountered, and constructive feedback to improve the program. Each patient was called and asked standardized questions about their experience. They were also invited to participate in a focus group interview and to offer non-scripted feedback.

Analysis

All Likert responses were tabulated as counts and percentages, with 95% confidence intervals (95% CI) measured between SDOH metrics. Continuous data were compared using paired Student's T-tests, with p < 0.05 as significant.

## Results

Of the 60 OUD patients initiated on MOUD during the study period, 19 (31.7%) were included, of which 16 (84%) completed all the survey questions. Survey participants ranged in age from 30 to 64 years, with a mean age of 42 years, which was similar to the 41 patients who did not complete the survey (mean age = 41 years). All but one had White ethnicity (94%), which is slightly higher than the nonresponders (88%) (p = 0.24). Thirteen of the 19 surveyed patients identified as male (68%) and six as female (32%); similar to the 41 nonresponders (64% male and 36% female, p = 0.33). Of the survey responders, 73% had a high school degree or higher, which was identical to the nonresponders (73%). Of the survey responders, 19% were employed, which was slightly lower than the nonresponders (28%) (p = 0.16). The zip code analysis noted a higher median income in the study population ($57,080) compared to the surrounding county ($45,534) (p < 0.001).

Survey results regarding the ACC were largely positive, with 93% of those surveyed viewing the ACC as "Helpful" or "Very helpful" (95% CI: 91-95%). Participants reported the ACCs to be especially helpful in coordinating post-ED addiction care, organizing peer coach involvement, prompting engagement and treatment during ED visits, and addressing and overcoming financial and social barriers to MOUD (Table 1).

**Table 1:** Survey results on ACC utilization. 1 = not helpful; 5 = very helpful. ACC = addiction care coordinator.

ACC usefulness	1	2	3	4	5
Coordinating initial follow-up appointment	6.3%	0.0%	0.0%	6.3%	87.4%
Connecting to community resources	6.3%	0.0%	6.3%	12.5%	74.9%
Discuss the overall recovery process	6.3%	0.0%	0.0%	6.3%	87.4%
Kept engaged in the treatment program	12.5%	0.0%	12.5%	12.5%	62.5%
Assisting with overcoming social barriers	6.3%	0.0%	6.3%	12.5%	74.9%
Instructing on technology use	12.5%	0.0%	0.0%	6.3%	81.2%

Over 90% (95% CI: 87-93%) felt comfortable with using telemedicine treatment for their OUD treatment. All 60 patients had access to cell phones, including the 19 who participated in the survey. Survey respondents also felt the ACC helped them gain control of their lives (63% strongly agreed; 95% CI: 59-67%) and improved their overall health (57% agreed their health was better; 95% CI: 53-61%).

Respondents answered questions regarding barriers they experienced in obtaining care (Table 2).

**Table 2:** SDOH survey. 1 = almost always; 5 = almost never. SDOH = social determinants of health.

SDOH issues	1	2	3	4	5
Transportation issues	12.5%	0.0%	25.0%	0.0%	62.5%
Follow-up appointments	0.0%	0.0%	18.8%	0.0%	81.2%
Technology use	12.5%	0.0%	12.5%	0.0%	75.0%
Financial issues	12.5%	0.0%	25.0%	12.5%	50.0%
Ability to get prescriptions filled	6.3%	0.0%	6.3%	0.0%	87.4%

Transportation to medical appointments and financial issues were the most prevalent social barriers to remaining in treatment (p = 0.0073). Almost all of the unsolicited comments were positive about the program. Several commented on the well-rounded and discreet care coordination, while others praised the ease of the telemedicine component.

## Discussion

OUD is a significant health concern for Ohio and the nation [3]. Opioid use in the population continues to increase. It is imperative that we develop programs to help people recover. Health disparities occur when these groups experience more disease, death, or disability beyond what would normally be expected based on their relative size of the population. Health disparities are often characterized by such measures as disproportionate incidence, prevalence, and/or mortality rates of diseases or health conditions. Health is largely determined by where people live, work, and play. Health disparities are unnatural and occur because of low socioeconomic status, race/ethnicity, sexual orientation, gender, disability status, geographic location, or some combination of these factors [6]. Those most impacted by health disparities also tend to have less access to resources like healthy food, safe housing, quality education, safe neighborhoods, and freedom from racism and other forms of discrimination [7]. These are referred to as SDOH. SDOH are one of the root causes of health disparities [14]. Our focus group survey concentrated on those SDOH that affected care and recovery in our ED-based MOUD program. We used the metrics from the HOPE initiative [11] and SHIP [12] to develop the questions in our focus survey, as mandated by our ODH grant. The survey concentrated on mental health recovery questions and social and economic factors such as housing, education, transportation, employment, and access to psychiatric care. We also wanted to ascertain whether virtual services were helpful in this population. If found to be effective, the ability to provide virtual services greatly expands access to reduce healthcare disparities.

An examination of the existing literature shows that socioeconomic factors underlie OUD presentations. Socioeconomic barriers do not only predispose people to use drugs but health disparities have also been noted in minority groups who are opioid users [15,16]. Studies have shown that delayed hospital presentation has an impact on the outcomes of diverse medical issues. Social factors contribute to the delay in seeking care for several medical illnesses, including those requiring acute treatments [15,16]. Moreover, they have also been associated with a higher risk of hospitalization, mortality, and extended stay [15]. Socioeconomic status is essential as we look at OUD to reflect cause and effects [16]. Hence, there is a dire need to understand rural and urban socioeconomic factors about varied rural-urban OUD presentations [17].

Understanding the diverse influences underlying the onset and maintenance of OUD is necessary for effective prevention and intervention strategies. Evidence suggests that optimizing SDOH as part of effective treatment may positively impact the lives and health outcomes of some with OUD. Furthermore, in a large longitudinal prospective cohort study of 615 heroin users, greater time spent in treatment for OUD was associated with improvements in aspects of SDOH, such as criminality, psychopathology, and mental health [18,19].

Our ACCs provided a service to the MOUD surveyed patients that they highly valued. The vast majority of participants indicated the ACCs were helpful in keeping them engaged in treatment, making it easy to contact, coordinating follow-up, and connecting them to other resources. The ACCs also provided a private, in-depth clinical interview with patients to express any safety concerns, particularly allowing for the opportunity to evaluate victims of human trafficking. As OUD is a significant health concern, EDs must develop programs to help people recover. Those most impacted by health disparities also tend to have less access to resources [15,16]. The focus group survey concentrated on those SDOH that affected care, including mental health recovery questions and social and economic factors such as housing, education, transportation, employment, and access to psychiatric care.

Secondly, most of our surveyed patients did not have issues related to securing transportation, scheduling follow-up appointments, and obtaining and filling prescriptions. For those who experienced barriers with scheduling appointments or obtaining transportation, less than half indicated that Summa was able to assist them. This is an opportunity for improvement in our MOUD program.

Third, regarding the option and use of telemedicine for virtual consultations, about half of the surveyed respondents appreciated the option and found it useful, and about half indicated it was not applicable. This tells us that, if practical, providing virtual services could expand access and potentially reduce healthcare disparities.

Finally, well over half of the survey participants experienced an improvement in their overall health. At the same time, those in the program felt they had gained some control over their life and felt their treatment plan was effective in dealing with their OUD.

The telephone surveys and focus groups confirmed what ED clinicians intuitively know: the MOUD program in general and the work of the ACCs in particular help patients with OUD recover from their disease. The program can improve by ensuring barriers to getting transportation to appointments and filling prescriptions are addressed and solved. Patients appreciate the option of telemedicine consults, so we need to ensure that option is available. Finally, the telephone surveys and focus groups provide encouragement to all the providers that we are, in fact, helping these very sick and very vulnerable patients improve their health and lives.

There were several limitations of this study. As this study was part of a larger ODH grant, there were several specific requirements of the grant that affected the results. First, the grant had to be completed within the one-year timeframe. This survey needed to be completed so that six-month follow-up could be measured, hence the survey period was short. This may have affected internal validity. Secondly, the grant required the use of the HOPE and SHIP plans to address SDOH. This requirement affected the composition of our survey questions; however, the HOPE and SHIP plans were comprised of previously validated questions. We did not validate or measure the reliability of our survey. The small number of survey patients enrolled reflects the problematic nature of these patients in terms of follow-up and medical noncompliance [20]. The study was conducted at a single institution comprised of two EDs and two free-standing EDs. We also have a sophisticated MOUD program that utilizes ACCs for care coordination. Although this is deemed a best practice by the Substance Abuse and Mental Health Services Administration (SAMHSA), it is difficult to replicate at most institutions, affecting external validity.

## Conclusions

This study demonstrated the importance of collecting data on patient perspectives to inform programmatic strategy. From the individual interviews and focus groups, the study ascertained information related to a significant strength of the program and the value of the ACCs' role. Opportunities to improve the program included scheduling appointments and transportation. Evaluating SDOH will allow us to design program changes and interventions that address barriers, improve patient experience, and help patients stay in recovery.

## Figures and Tables

**Figure 1:**
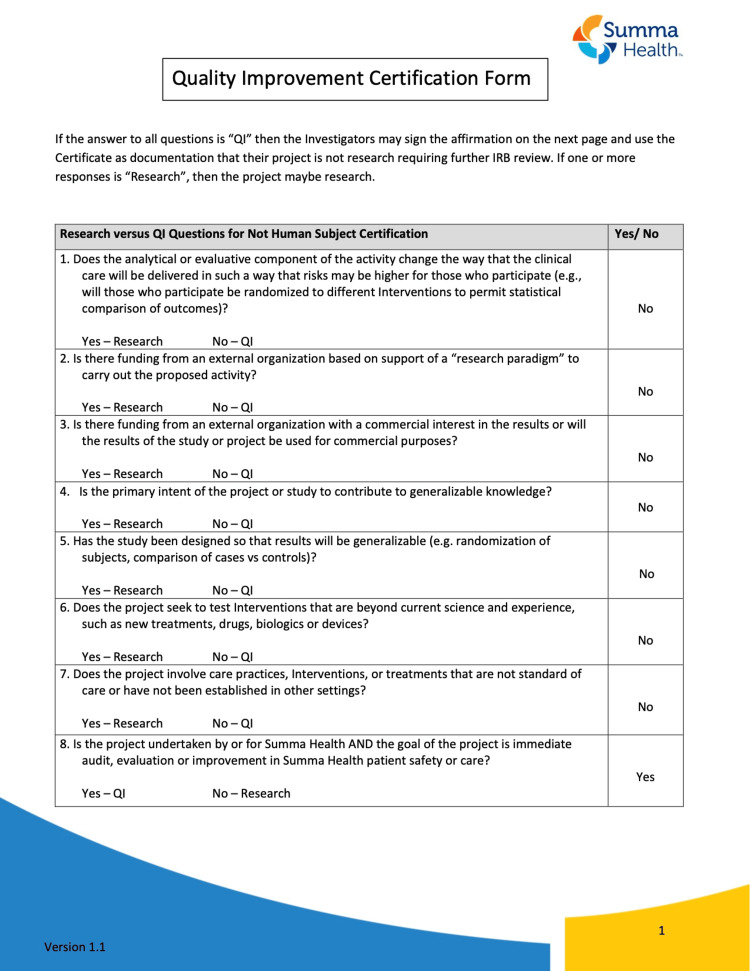
Page 1 of Summa Health IRB quality improvement form.

**Figure 2:**
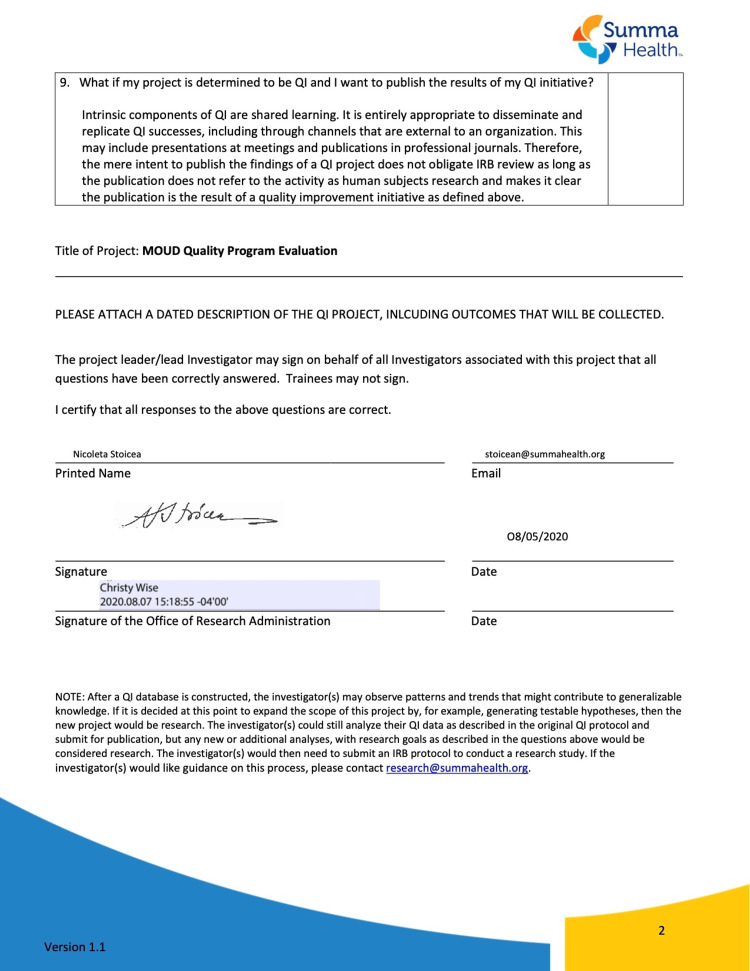
Signature page of Summa Health quality improvement form.
